# Energy-Based Approach to Predict Fatigue Life of Asphalt Mixture Using Three-Point Bending Fatigue Test

**DOI:** 10.3390/ma11091696

**Published:** 2018-09-12

**Authors:** Yazhen Sun, Chenze Fang, Jinchang Wang, Zuoxin Ma, Youlin Ye

**Affiliations:** 1 School of Transportation Engineering, Shenyang Jianzhu University, Shenyang 110168, China; fangchenze@126.com (C.F.); 15541947325@163.com (Z.M.); yeyoulin1985@126.com (Y.Y.); 2 Institute of Transportation Engineering, Zhejiang University, Hangzhou 310058, China

**Keywords:** energy-based approach, dissipated strain energy, plateau value of dissipated strain energy ratio, fatigue life, three-point bending fatigue test

## Abstract

The three-point bending fatigue tests were carried out in order to accurately predict the fatigue life of an asphalt mixture based on the plateau value (PV) of the dissipated strain energy ratio (DSER). The relations of the dissipated strain energy (DSE) to the stress-strength ratio, temperature and loading rate were studied, and the constructions of the mathematical models of DSE and DSER were completed based on the change laws of the DSE. The relation of the fatigue life to the PV was determined based on the analysis of damage evolution, based on which the fatigue equation was established and used to predict the fatigue life. The results show that the change laws of DSE and DSER can be well described by the proposed mathematical models. The PV is defined as the average value of the DSER in the second stage and the fatigue life decreases in power function with the increase of PV, based on which the fatigue equation of *N*_f_ = *A*(PV)*^B^* was established, and the established fatigue equation is very close to that is used in the MEPDG. The fatigue equation can well predict the fatigue life asphalt mixture.

## 1. Introduction

In recent years, more and more asphalt pavements have been built, but the qualities of part of them are not guaranteed. The asphalt pavement, subjected to repeated actions of different kinds of loads, is prone to fatigue failure, because of the strength reduction and the fatigue damage of materials [1]. The strength of asphalt mixture decreases gradually with the increase of the cyclic loading times, and the attenuated material strength can be used to define the damage. Some damage models were proposed to study the fatigue performance of asphalt mixture based on the change laws of the strength reduction [2,3]. The bearing capacity of asphalt mixture decreases with the increase of damage. The strength reduction will lead to the damage and the damage evolution, and the damage evolution accelerates the strength reduction [4,5]. When the damage evolves to the threshold of the failure, the bearing capacity will be less than the applied load, and the fatigue failure will occur to the material structure [6,7].

Many researches have been carried out to study the influence of experimental factors on the fatigue resistance of asphalt mixture. Furthermore, many mathematical models (such as the mathematical models of recovery ratio of elastic deformation, permanent deformation and permanent deformation ratio, etc.) were proposed to study the fatigue resistance of asphalt mixture, and it was found that the permanent deformation ratio reflects the damage evolution speed and can be well used to predict the fatigue life [8]. Logarithmic fatigue life linearly decreases with the increase of stress-strength ratio, and the fatigue life at low frequency is much less than that at high frequency [9]. There are many researches about the effects of the asphalt type on the fatigue life were carried out by experts. Three kinds of asphalt were used to study the effects of the asphalt type on the fatigue life through the fatigue test, and the results show that the rubber asphalt can well improve the fatigue performance of asphalt mixture compared with the base asphalt, styrene butadiene styrene (SBS) modified asphalt [9]. When the bitumen aggregate ratio is between 7.5% and 9%, the fatigue life of the rubber asphalt mixture increases with the increase of asphalt content [10]. The fatigue life of the rubber asphalt mixture increases with the decrease of the air voids in the reasonable range [11]. The high fatigue life of the rubber asphalt mixture was found to be closely related to the gradation [12]. Three kinds of gradation were used to study the effects of the asphalt mixture type on the fatigue life through the fatigue test, and the results of the research show that when the stress-strength ratio is relatively low (0.3–0.5), the gradation has a significant effect on the fatigue life of the asphalt mixture, and the increasing sequence of the fatigue life of different asphalt mixture types corresponds to the asphalt mixture types of AC-13, stone mastic asphalt (SMA), and gap gradation, respectively, which was caused by the reason that the internal structure and the air voids are different when the gradation changes. However, when the stress-strength ratio is relatively high (0.6–0.8), the gradation has no obvious effect on the fatigue life of the asphalt mixture [9]. 

The energy (damage) approach is widely used in the fatigue-life prediction, because of its simple principle and convenient operation [13,14,15,16]. The energy dissipation law of asphalt mixture was studied and the viewpoint that the dissipated strain energy (DSE) of material is related to their fatigue properties was proposed by Heukelom [17]. The total amount of DSE before the fracture of specimen was analyzed and the viewpoint that the maximum number of loading cycles can be affected by the cumulative DSE was proposed by Chomton and Valayer [18]. The viewpoint that there is a power function relation between the fatigue life and the cumulative DSE of the material structure before its fatigue failure was proposed based on the assumption that all the DSE before failure can cause the damage of material by Van Dijk [19]. The conclusion that temperature and loading mode can affect the mathematical relationship between the fatigue life and cumulative DSE of asphalt mixture was proved by Tayebali [20]. The damage evolution process was studied from the angle of energy by using the change laws of DSE and recoverable strain energy based on the different viscoelastic properties of asphalt mixture in the tensile and compressive portion by Xue Luo [21].

Most of the above researches on the fatigue-life prediction for asphalt mixture, based on the assumption that all the DSE before failure can cause the damage of material, cannot accurately reveal the damage evolution mechanism. In fact, only partial DSE can cause the fatigue damage to asphalt mixture, and the change of DSE is the real cause of material damage [22,23,24,25,26]. Therefore, the damage variable, defined by the cumulative total amount of dissipative energy before the fatigue failure, cannot accurately reveal the damage evolution process of asphalt mixture. The fatigue equation based on the damage variable cannot be used to correctly predict the fatigue life before fatigue failure.

In order to accurately reveal the damage evolution process and accurately predict the fatigue life of asphalt mixture, three-point bending fatigue tests were carried out. The conclusion that the DSE becomes smaller, with the increase of fatigue life, was obtained by analyzing the influences of temperature, stress ratio and rate on the calculation results of DSE. The mathematical models of DSE and dissipated strain energy ratio (DSER) was established based on the change laws of the DSE. The conclusion that the fatigue life decreases with the increase of DSER was obtained by defining the damage variable using DSER and studying the damage evolution process. The fatigue equation, established based on the relation of the fatigue life to the plateau value (PV) of DSER, was used to predict the fatigue life, and the results show that it can accurately predict the fatigue life of asphalt mixture.

The applied main research methodology was that the fatigue test was used for the theoretical analysis of the relation of the fatigue life to the PV, and the fatigue equation was established based on the relation.

## 2. Materials and Experimental Procedures

The clamping structure of four-point bending fatigue test is complex, and the three-point bending fatigue test has the strong applicability and the simple loading mode. Therefore, the three-point bending fatigue test was used to study the fatigue life of asphalt mixture in this paper [27].

### 2.1. Test Materials

The 70# rubber modified asphalt (marking in accordance with the Chinese standard of the Technical Specifications for Construction of Highway Asphalt Pavements (JTGF40-2004)) with a penetration of 70 produced from Ningbo was used as asphalt binder and its specifications provided by the manufacturer are listed in Table 1 [28]. The ratio of binder to aggregate is 8.8% by weight. The limestone was used as the aggregate, and the continuous aggregate gradation type is AC-13 as listed in Table 2, has the nominal maximum aggregate size of 13.2 mm. The AC-13 asphalt mixture is widely built in the surface course of highway with the standard axle load of 100 kN designed by the specifications for design of highway asphalt pavement (JTGD50-2017) [29].

### 2.2. Preparation of Specimen

The temperature of aggregates and asphalt was 160 °C, and the temperature of compaction was 100 °C. The sample forming machine was used to form the rut board (400 mm × 400 mm × 70 mm). The rut board was cut to obtain the specimen beams (250 mm × 30 mm × 35 mm) with the average density of 2.445 g/cm^3^ and the size error of the specimen beams should be controlled within 2 mm [30]. 

### 2.3. Test Conditions and Methods

The ratio of the peak stress of each cycle to the ultimate material strength is called the stress-strength ratio (SSR). The fatigue tests had three groups of contrast tests in order to study the relationship between the fatigue life and the experimental factors. In order to reduce the test error, three parallel specimens, that is, a total of 18 specimens were selected for the fatigue test:

Group 1. For this group, the temperature was 25 °C, the loading rates were 10 mm/min and 20 mm/min, respectively, and the SSR was 0.6.
Group 2. For this group, the temperature was 5 °C, 15 °C, and 25 °C, respectively, the loading rate was 10 mm/min, and the SSR was 0.6.
Group 3. For this group, the temperature was 25 °C, the loading rate was 10 mm/min, and the SSRs were 0.6, 0.7, and 0.8 respectively.

### 2.4. Test Results 

During each loading cycle, the specimen was loaded linearly until the deformation reached the peak and was unloaded linearly until the stress reached zero. The fatigue life was determined by the loading number corresponding to the apparent fracture of the specimen which loses the carrying capacity.

The 18 specimens were tested according to the test scheme and the average fatigue life of the 3 parallel specimens was taken as the final fatigue life. The statistical results of the fatigue lives are listed in Table 3, from which it can be seen that each coefficient of variation is less than 8.15%. The fatigue life decreases with the increase of the SSR and the loading rate, and increases with the increase of the temperature, form which the spread conclusions that the fatigue resistance of asphalt pavement can be improved by properly reducing the axle load and the driving speed can be drawn.

## 3. Construction of DSE Mathematical Model

**Figure 1:**
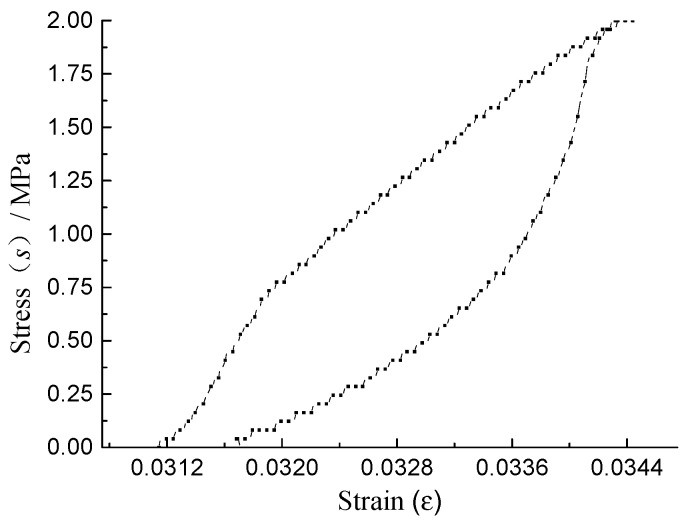
Stress-strain curve.

Applying a stress to a material will induce a strain. The energy being input into the material is represented by the area under the stress-strain curve of three-point bending fatigue test. The strain will recover when the stress is removed from the material, as shown in Figure 1. If the loading and unloading curves coincide, all the energy put into the material is recovered or returned after the load is removed. If the two curves do not coincide, there is energy lost in the material, energy that was dissipated through mechanical work, heat generation, or damage to the material in such a manner that it could not be used to return the material to its original shape. This energy difference is the dissipated energy of the material caused by the load cycle.

### 3.1. Influence of Experimental Factors on DSE

**Figure 2:**
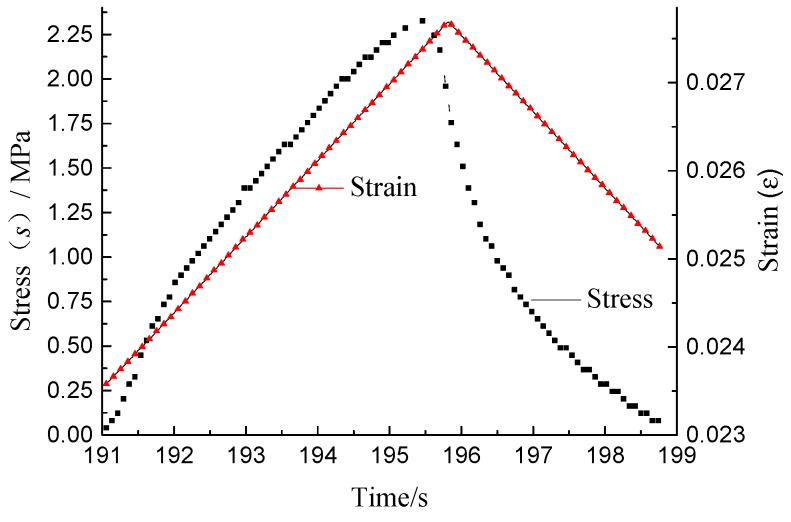
Change curves of stress and strain verses time.

During each loading cycle, the asphalt mixture specimen was loaded linearly at the given rate until the deformation reached the peak in the loading stage; and was unloaded linearly at the given rate until the deformation reached zero in the unloading stage. The curves of stress-time and strain-time of each loading cycle are shown in Figure 2, from which it can be seen that the time corresponding to the strain peak of asphalt mixture is later than it to the stress peak, because of the viscoelastic hysteresis characteristics of asphalt mixture. As shown in Figure 1, the stress-strain curve of each loading cycle before the fatigue fracture are hysteretic closed curve, because of the viscoelastic hysteresis characteristics and the internal area of the curve is equal to the DSE in each loading cycle. The DSE of the loading cycle *N* (DSE*_N_*), caused by the irrevocable deformation, is composed of the ${\mathrm{DSE}}_{N}^{\vartheta }$ and the ${\mathrm{DSE}}_{N}^{\varepsilon }$. The ${\mathrm{DSE}}_{N}^{\varepsilon }$, used to produce the damage deformation of the asphalt mixture, is closely related to the fatigue life, and ${\mathrm{DSE}}_{N}^{\vartheta }$, used to produce the viscoelastic deformation, is independent of the fatigue life.

(1) $${\mathrm{DSE}}_{N}^{}={\mathrm{DSE}}_{N}^{\vartheta }+{\mathrm{DSE}}_{N}^{\varepsilon }\;$$

where ${\mathrm{DSE}}_{N}^{\vartheta }$ is the DSE used to produce the viscoelastic deformation of the loading cycle *N*, and ${\mathrm{DSE}}_{N}^{\varepsilon }$ is the DSE used to produce the damage deformation of the loading cycle *N.* The ${\mathrm{DSE}}_{N}^{\vartheta }$ of the loading cycle *N* is a fixed value under certain condition which contains temperature, stress ratio and loading rate [22].

**Figure 3:**
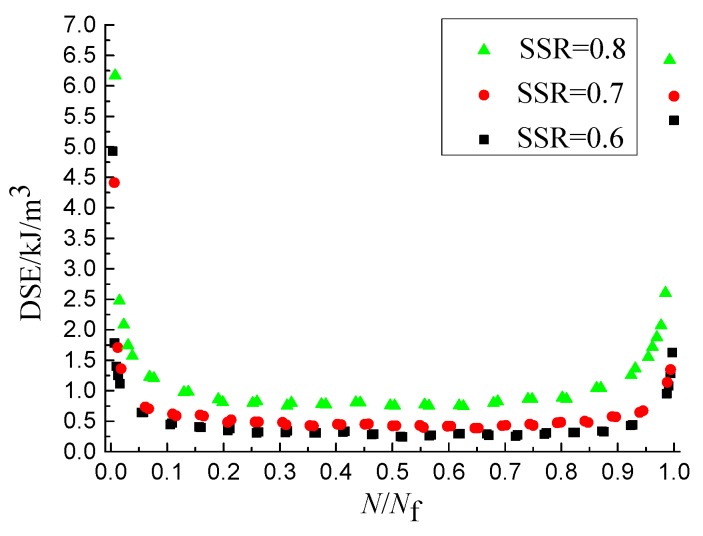
The influence of SSR on dissipated strain energy (DSE).

**Figure 4:**
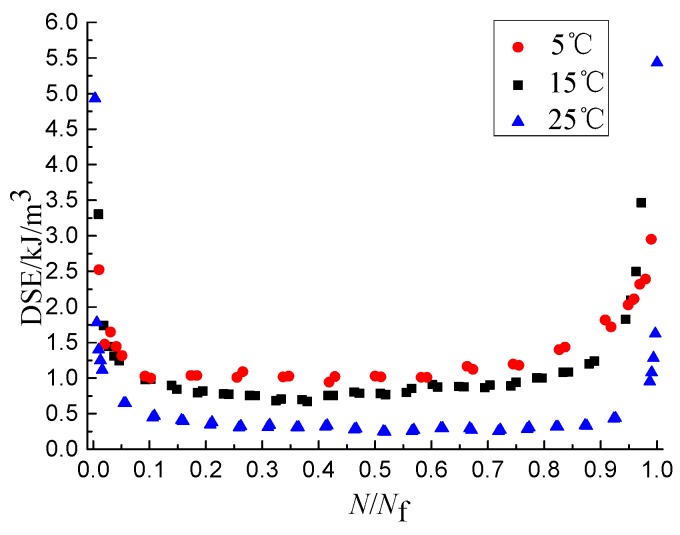
The influence of temperature on DSE.

**Figure 5:**
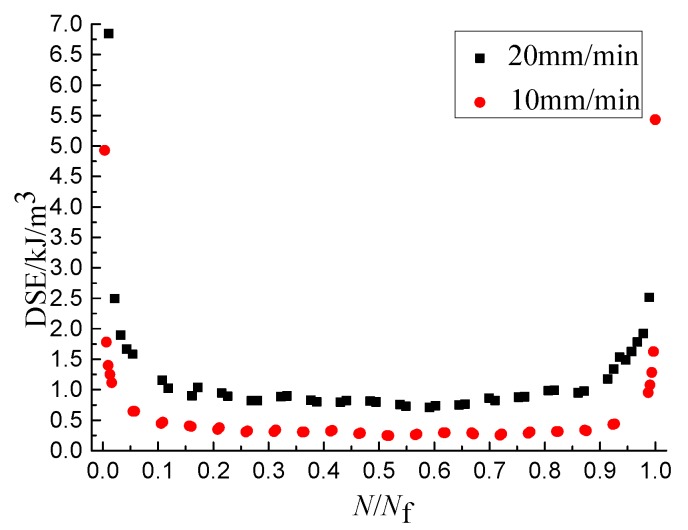
The influence of loading rate on DSE.

The DSE*_N_* is obtained by calculating the internal area of the closed curve using the tool of ORIGIN. The abscissa is converted into dimensionless quantities, and the DSE-*N*/*N*_f_ curves are shown as Figure 3, Figure 4 and Figure 5, from which it the can be seen that the DSE-*N*/*N*_f_ curves can be divided into three stages and the overall shape looks like the shape of “U”. The DSE of the first stage decreases rapidly, but lasts a short time, and the value of the DSE is small at the end of the first stage. The DSE of the second stage changes stably, but lasts a long time. The DSE of the third stage increases rapidly. As shown in Figure 3, Figure 4 and Figure 5, the DSE increases with the increase of SSR and loading rate and decreases with the increase of temperature. Therefore, the DSE decreases with the increase of the fatigue life. Because the stress amplitude and deformation of the single cycle specimen increases when the SSR increases, and the specimen with the same force needs greater deformation when the temperature decreases or the loading rate increases. This causes the internal structure of asphalt mixture dissipate more energy to complete the recompositing.

### 3.2. Construction of DSE Mathematical Model

**Figure 6:**
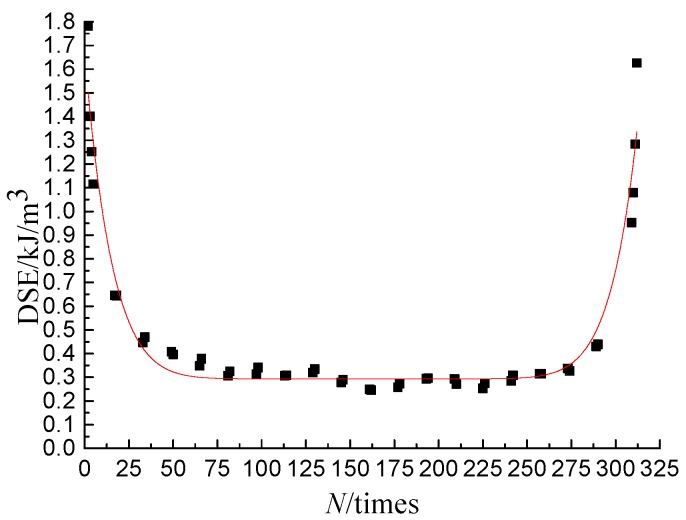
Fitting effect of DSE-*N* curve.

In order to describe the change law of DSE accurately, the DSE mathematical model, shown in Equation (2), was proposed by studying the change of the DSE verses the number of loading cycles. The DSE mathematical model was used to fit the DSE-*N* curve, and the fitting effect is shown in Figure 6, with the correlation coefficient greater than 0.95, which indicates that the change law of DSE can be well described by the model. The fitting results of DSE-*N* curves of different test conditions are listed in Table 4, from which it can be seen that the value ranges of *c*_1_–*c*_4_ are 0.110–0.554, 0.013–0.122, 0.269–1.134 and 0.118–1.878, respectively.

(2) $$\mathrm{DSE}(N)=c_{1}+c_{2}\left|{\left(N-c_{3}\right)}^{c_{4}}\right|,$$

where *c*_1_, *c*_2_, *c*_3_ and *c*_4_ are fitting parameters.

**Figure 7:**
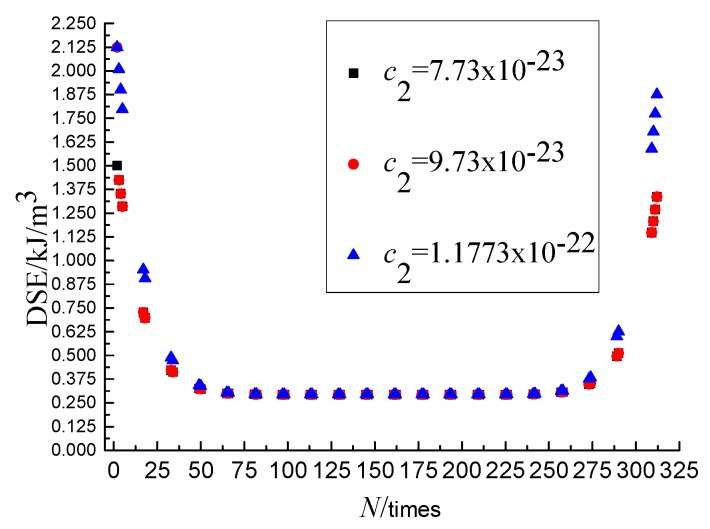
Sensitivity analysis of parameter *c*_2._

**Figure 8:**
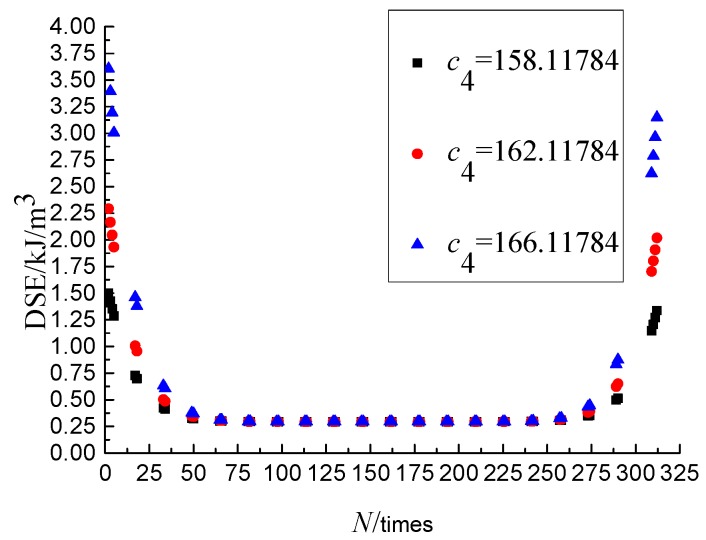
Sensitivity analysis of parameter *c*_4._

Obviously, the overall calculation result of DSE and the left and right translation of graph can be affected by the *c*_1_ and *c*_3_, respectively. This section takes the parameters obtained at the SSR of 0.6, the loading rate of 10 mm/min, and the temperature of 25 °C as an example to analysis the sensitivity of *c*_2_ and *c*_4_. The *c*_1_ and *c*_3_ keeping invariant, set the values for the *c*_1_ as 7.7304 × 10^−23^, 9.7304 × 10^−23^ and 1.177304 × 10^−23^, respectively, and the *c*_3_ as 158.11784, 162.11784, 166.11784, by which the DSE-*N* curves were drawn, as shown in Figure 7 and Figure 8, from which it can be seen that the change of DSE in the second stage is not affected and the DSE in the first and third stage are affected by the *c*_2_ and *c*_4_.

The parameters *c*_1_–*c*_4_ are obtained by fitting the DSE mathematical model, and each parameter has the specific physical meaning. From the above analysis, we can draw the conclusion that the c_1_ is the major parameter that affects the overall calculation result of DSE, and the *c*_3_ is the major parameter that affects the left and right translation of graph. The *c*_2_ and *c*_4_ are the main parameters affecting the change of DSE in the first and the third stage.

## 4. Construction of DSER Mathematical Model

The DSE is gradually accumulated during the damage evolution. At present, the study of fatigue damage evolution based on DSE is mostly based on the assumption that all the DSE causes damage to asphalt mixture. The assumption has been proved to be inaccurate and damage to the asphalt mixture will be caused by the change of DSE [31]. The concept of a dissipated strain energy ratio (DSER) was proposed based on the absolute value of the change in dissipated strain energy between load cycle *N* and load cycle *N* + 1 divided by the dissipated strain energy in load cycle *N* [22,32]:

(3) $$\mathrm{DSER}=\frac{\left|{\mathrm{DSE}}_{N+1}^{}-{\mathrm{DSE}}_{N}^{}\right|}{{\mathrm{DSE}}_{N}},$$

where DSE*_N_*_+1_ and DSE*_N_* are the DSE of the loading cycle *N* + 1 and *N*. The DSER represents the percentage of dissipated energy causing damage to the material. 

**Figure 9:**
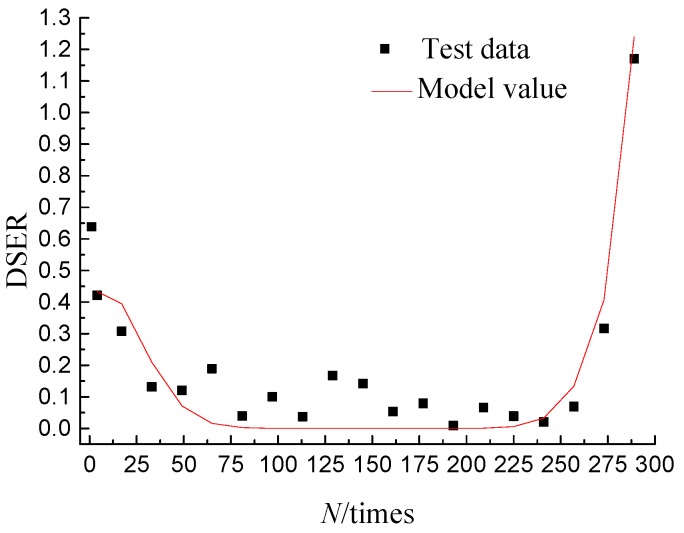
Contrast result of dissipated strain energy ratio (DSER).

The ${\mathrm{DSE}}_{N}^{\vartheta }$ of the loading cycle *N* is a fixed value under certain conditions, therefore, the proportion of the ${\mathrm{DSE}}_{N}^{\varepsilon }$ to the DSE*_N_* is reflected by the DSER that is only related to the damage deformation [22,23]. Mastering the change law of DSER can provides a theoretical basis for the study of damage evolution. In order to accurately describe the change law of DSER in the process of damage evolution, the DSER mathematical model of the three-point bending fatigue test, obtained by substituting the Equation (2) into the Equation (3), is shown in Equation (4). The DSER results of the mathematical model were used to compare with that obtained by tests, and the comparison results are shown in Figure 9, from which it can be seen that the mathematical model well predict the overall change trend of DSER.

(4) $$\mathrm{DSER}(N)=\frac{\left|{\mathrm{DSE}}_{N+1}^{}-{\mathrm{DSE}}_{N}^{}\right|}{{\mathrm{DSE}}_{N}^{}}=\frac{c_{2}\left|\left|{\left(N+1-c_{3}\right)}^{c_{4}}\right|-\left|{\left(N-c_{3}\right)}^{c_{4}}\right|\right|}{c_{1}+c_{2}\left|{\left(N-c_{3}\right)}^{c_{4}}\right|}\;$$

## 5. Fatigue-Life Prediction Based on the Energy Approach

### 5.1. Analysis of Damage Evolution Based on the DSER

DSE is used to produce damage and viscoelastic deformation, and the DSER reflects the proportion of the DSE that produces the damage deformation to the total DSE. The DSER is only related to the damage deformation, so it was used to analyze the damage evolution. The damage factor can be expressed based on the DSER as,

(5) $$D=\frac{\sum_{k=1}^{N}{\mathrm{DSER}}_{k}}{\sum_{k=1}^{N_{f}}{\mathrm{DSER}}_{k}}\;$$

where DSER*_k_* is the DSER at the loading cycle *k*, and *D* is the damage factor.

**Figure 10:**
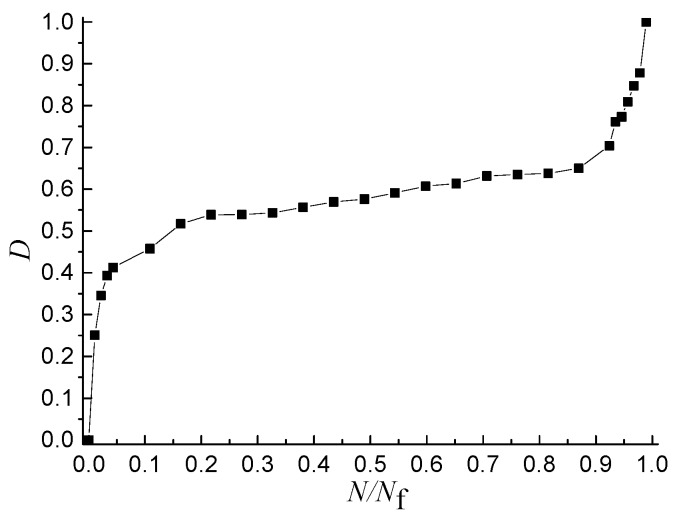
*D*-*N*/*N*_f_ curve.

**Figure 11:**
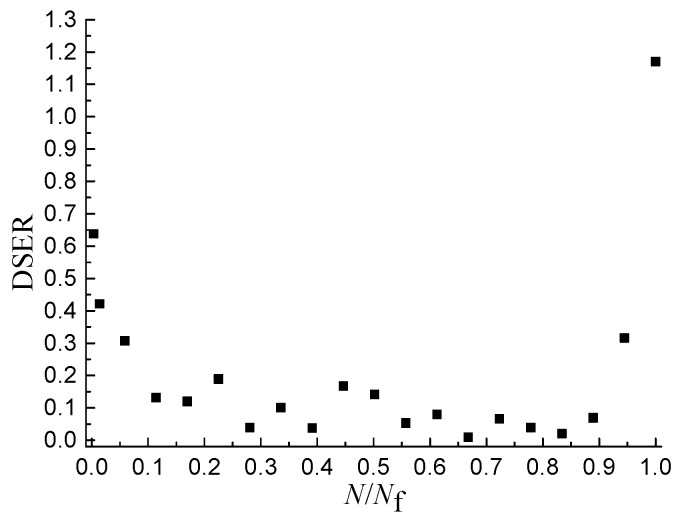
Scatter plots of DSER-*N*/*N*_f._

In order to study the relationship between the DSER and the damage evolution, the damage evolution process reflected by the *D*-*N*/*N*_f_ curve (Figure 10) and the DSER changing process reflected by the *DSER*-*N*/*N*_f_ scatter plot (Figure 11) was analyzed, respectively.

The nonlinear damage evolution process can be divided into three stages, as shown in Figure 10, and the proportion of the duration of the first stage to that of the whole process is less than 5%, but the damage value at the end of the first stage is greater than 0.4. The damage of the second stage increases stably, but the proportion of the duration of the first stage to that of the whole process is around 90%, and the damage of the third stage increases sharply until the specimen fractures. 

The DSER changing process can be also divided into three stages and the proportion of the duration of each stage to that of the whole changing process is close to that of the damage evolution process, as shown in Figure 11. In the first stage, the DSER value is greater than 0.3, but the DSER decreases gradually and the slope of the descent curve gradually decreases. The DSER value of the second stage is less than 0.2, but the whole trend approaches a stable value. The DSER of third stage increases sharply in a short period of time.

The damage evolution speed and the DSER values in the first and third stages is far greater than that in the second stage [33], from which it can be seen that the three stages of the DSER evolution and the damage evolution correspond to each other, and there is a positive correlation between the damage evolution speed and the DSER. The DSER can be used as the energy parameter to characterize the damage evolution speed. The speed of the damage closing to failure threshold increases with the increase of the damage evolution speed. Therefore, the fatigue life decreases with the increase of the DSER.

### 5.2. Establishment of Fatigue Equation Based on the PV of DSER

The DSER changing process can be divided into three stages. Although the calculation results of DSER in the first and third stages are greater than that of the second stage, the second stage is the main part of the whole process which accounts for more than 80% of the whole process. Therefore, the DSER value of second stage reflects the overall DSER value of the three stages. The plateau value (PV) of DER represents a period during which there is a constant percentage of input energy being turned into damage, and as such may prove to be a material property useful in design [32]. Therefore, in this paper, the plateau value (PV) is defined as the average value of DSER in the second stage, and PV reflects the overall DSER value of the three stages and the resistance to fatigue damage.

The results of the PV and the fatigue life (*N*_f_) of the asphalt mixture are listed in Table 5, which indicates that *N*_f_ decreases with the increase of PV. The power function with the advantages of simple form and easy generalization was used to establish the fatigue equation as shown below, in Formula (6). The fatigue equation was used to fit the results in Table 5, and the fitting results are shown in Table 6. The correlation coefficient is greater than 0.95, which proves the reliability of the fatigue equation used to calculate the predicted fatigue lives. The parameter *B* is shown as −3.29 in the Table 6, this is very close to −3.95 that is used in the MEPDG (AASHTO Ware ME Design), thus the research is consistent with known results [34]. The comparison between the predicted life and the test life is listed in Table 7, which indicates that the fatigue life can be well predicted by the fatigue equation, based on the PV of DSER.

(6) $$N_{f}=A{\left(\mathrm{PV}\right)}^{B},$$

where *A* and *B* are fitting parameters.

## 6. Conclusions

(1) The DSE increases with the increase of the SSR and the loading rate, and the decrease of the temperature. That is, the fatigue life of the asphalt mixture decreases with the increase of the DSE.
(2) The change laws of DSE and DSER verses the number of loading cycles can be well described by the proposed mathematical models.
(3) The DSER is only related to the damage deformation. The speed of the damage closing to failure threshold increase with the increase of the damage evolution speed and the fatigue life decreases with the increase of the DSER.
(4) The fatigue life decreases in power function with the increase of PV, based on which the fatigue equation was established. The established fatigue equation is very close to that is used in the MEPDG (AASHTO Ware ME Design). The fatigue equation can well predict the fatigue life asphalt mixture.

## Figures and Tables

**Table 1:** Properties of asphalt rubber.

Properties	Standard	Value
Penetration (25 °C,100 g, 5 s)	T0604-2011	70 (0.1 mm)
Softening point	T0606-2011	57 (°C)
Viscosity (177 °C)	T0625-2011	3.8 (Pa·s)
Elastic recovery (25 °C)	T0662-2011	72 (%)

**Table 2:** Aggregate gradation.

Sieve Size (mm)	16.0	13.2	9.5	4.75	2.36	1.18	0.6	0.3	0.15	0.075
Passing percentage	100.0	91.1	80.2	54.0	33.2	22.5	16.0	12.1	8.7	5.5

**Table 3:** Fatigue lives.

Stress-Strength Ratio (SSR)-Temperature-Loading Rate	Sample 1	Sample 2	Sample 3	Average Value	Coefficient of Variation (%)	Standard Deviation
0.6–25 °C-10 mm/min	329	312	298	313	4.05	12.68
0.7–25 °C-10 mm/min	168	150	165	161	4.89	7.87
0.8–25 °C-10 mm/min	140	128	149	139	6.19	8.60
0.6–15 °C-10 mm/min	105	113	109	109	3.00	3.27
0.6–5 °C-10 mm/min	91	101	99	97	4.45	4.32
0.6–25 °C-20 mm/min	120	119	100	113	8.14	9.20

**Table 4:** Fitting parameters of DSE-*N* curve.

SSR-Temperature-Loading Rate	*c*_1_	*c*_2_	*c*_3_	*c*_4_	*R* ^2^
0.6–25 °C-10 mm/min	0.29359	7.7304 × 10^−23^	158.1178	10.1179	0.9512
0.7–25 °C-10 mm/min	0.44862	2.0989 × 10^−19^	83.74164	9.79295	0.9606
0.8–25 °C-10 mm/min	0.79304	5.6107 × 10^−16^	65.24516	8.55627	0.9685
0.6–15 °C-10 mm/min	1.01016	3.1166 × 10^−7^	42.66207	3.83088	0.9059
0.6–5 °C-10 mm/min	0.8327	2.6332 × 10^−25^	52.42931	14.5094	0.9093
0.6–25 °C-20 mm/min	0.83581	3.9988 × 10^−15^	46.86074	8.81419	0.9622

**Table 5:** Statistical Results of *N*_f_ and plateau value (PV).

*N*_f_	PV
313	0.0209
164	0.02484
129	0.02592
106	0.02829
98	0.03065
92	0.0315

**Table 6:** Fitting Parameters of Fatigue Equation.

Fitting Parameters	*A*	*B*	*R* ^2^
**Results**	8.91785 × 10^−4^	−3.29402	0.95553

**Table 7:** Contrast results of fatigue life.

Stress Strength Ratio-Temperature-Loading Rate	Test Results of *N*_f_	Predictive Results of *N*_f_	Relative Error
0.6–25 °C-10 mm/min	313	305	2.5%
0.7–25 °C-10 mm/min	164	172	4.9%
0.8–25 °C-10 mm/min	129	150	16.3%
0.6–25 °C-20 mm/min	106	112	5.7%
0.6–15 °C-10 mm/min	98	79	19.4%
0.6–5 °C-10 mm/min	92	76	17.4%
